# Correction: Sustainable polymeric adsorbents for adsorption-based water remediation and pathogen deactivation: a review

**DOI:** 10.1039/d4ra90129k

**Published:** 2024-11-04

**Authors:** Huda Alkhaldi, Sarah Alharthi, Salha Alharthi, Hind A. AlGhamdi, Yasmeen M. AlZahrani, Safwat A. Mahmoud, Lamiaa Galal Amin, Nora Hamad Al-Shaalan, Waleed E. Boraie, Mohamed S. Attia, Samera Ali Al-Gahtany, Nadiah Aldaleeli, Mohamed Mohamady Ghobashy, A. I. Sharshir, Mohamed Madani, Reem Darwesh, Sana F. Abaza

**Affiliations:** a College of Science and Humanities, Jubail Imam Abdulrahman Bin Faisal University Jubail Saudi Arabia; b Department of Chemistry, College of Science, Taif University P.O. Box 11099 Taif 21944 Saudi Arabia; c Chemistry Department, College of Science, Imam Abdulrahman Bin Faisal University P.O. Box 1982 Dammam 31441 Saudi Arabia; d Physics Department, Faculty of Science, Northern Border University Arar Saudi Arabia; e Department of Chemistry, College of Science, Princess Nourah Bint Abdulrahman University P.O. Box 84428 Riyadh 11671 Saudi Arabia; f Department of Chemistry, College of Science, King Faisal University P.O. Box 400 Al-Ahsa 31982 Saudi Arabia; g Chemistry Department, Faculty of Science, Ain Shams University Abbassia Cairo 11566 Egypt; h Faculty of Science, University of Jeddah Jeddah Saudi Arabia; i Radiation Research of Polymer Chemistry Department, National Center for Radiation Research and Technology (NCRRT), Egyptian Atomic Energy Authority (EAEA) Cairo Egypt Mohamed_ghobashy@yahoo.com; j Solid State and Electronic Accelerators Department, National Center for Radiation Research and Technology (NCRRT), Egyptian Atomic Energy Authority (EAEA) Cairo Egypt; k Physics Department, Faculty of Science, King Abdulaziz University Jeddah Saudi Arabia; l Physics Department, Faculty of Science, Alexandria University 21568 Alexandria Egypt

## Abstract

Correction for ‘Sustainable polymeric adsorbents for adsorption-based water remediation and pathogen deactivation: a review’ by Huda Alkhaldi *et al.*, *RSC Adv.*, 2024, **14**, 33143–33190, https://doi.org/10.1039/D4RA05269B.

The authors regret that the name of one of the authors (Lamiaa Galal Amin) and one of the affiliations (affiliation *d*) were shown incorrectly in the original article. The corrected author list and affiliations are shown herein.

The Royal Society of Chemistry apologises for these errors and any consequent inconvenience to authors and readers.

